# Glioma Stem-Like Cells and Metabolism: Potential for Novel Therapeutic Strategies

**DOI:** 10.3389/fonc.2021.743814

**Published:** 2021-08-31

**Authors:** Abigail Harland, Xia Liu, Mattia Ghirardello, M. Carmen Galan, Claire M. Perks, Kathreena M. Kurian

**Affiliations:** ^1^Brain Tumour Research Centre, Bristol Medical School, University of Bristol, Bristol, United Kingdom; ^2^Galan Research Group, School of Chemistry, University of Bristol, Bristol, United Kingdom; ^3^IGFs and Metabolic Endocrinology Group, Bristol Medical School, Translational Health Sciences, Southmead Hospital, University of Bristol, Bristol, United Kingdom

**Keywords:** cancer stem cell (CSC), therapeutic strategies, cancer metabolism, glioma stem-like cell, metabolic reprogramming

## Abstract

Glioma stem-like cells (GSCs) were first described as a population which may in part be resistant to traditional chemotherapeutic therapies and responsible for tumour regrowth. Knowledge of the underlying metabolic complexity governing GSC growth and function may point to potential differences between GSCs and the tumour bulk which could be harnessed clinically. There is an increasing interest in the direct/indirect targeting or reprogramming of GSC metabolism as a potential novel therapeutic approach in the adjuvant or recurrent setting to help overcome resistance which may be mediated by GSCs. In this review we will discuss stem-like models, interaction between metabolism and GSCs, and potential current and future strategies for overcoming GSC resistance.

## Introduction

Innovative treatment approaches to Glioblastoma (GBM) have thus far been unsuccessful in part due to therapeutic resistance, resulting in disease recurrence (1). GBM is the most common intrinsic brain tumour in adults and is classed as the highest grade (IV) astrocytoma by the World Health Organisation (WHO) (2). Characteristic infiltration into surrounding structures of the brain as well as central necrotic regions can be identified using histopathological studies (2). Post diagnosis, GBM has a survival time of just 12-18 months in response to current chemoradiation protocols following surgical resection as outlined in the Stupp protocol (3–5). In addition, only around 5% of patients survive longer than 5 years post-diagnosis (5). Previously, GBMs have been categorised based on whether they derive from lower grade lesions, first defined by Scherer in the 1940s (2). The rapid and *de novo* development of aggressive lesions is defined as primary GBM, accounting for approximately 95% of cases and thought to develop in part from a defined set of oncogenic mutations (6). In contrast, secondary GBM cases have been identified as evolving from lower grade astrocytoma precursors, often distinguished by the presence of the isocitrate dehydrogenase 1 (*IDH1*) mutation and given a more favourable prognosis due to more frequent diagnosis in younger patients (6, 7).

To gain insight into the molecular drivers of GBM, studies have extensively profiled tumours, reporting both genetic and epigenetic mutations believed to play a part in tumour initiation and progression including loss of heterozygosity (LOH) 10q, amplification of epidermal growth factor receptor (EGFR), deletion of *p16INK4a* and mutations in tumour protein 53 (*TP53*) and phosphatase and tensin homolog (*PTEN*) (6). Reports from The Cancer Genome Atlas (TCGA) have also provided insight for grade IV tumours, providing a comprehensive understanding of genetic, expression and epigenetic aberrations (8). Clustering GBM data for gene expression, survival and treatment response has identified distinct neoplasm subtypes. The classification of these phenotypic profiles varies between research groups but include: proneural (PN), proliferative, sometimes split into neural (N) and classical (C) and mesenchymal (MES) (9, 10). Complicating matters further, data inclusive of intratumoural heterogeneity through genomic multisampling, has revealed the presence of multiple co-existing subtypes within the same patient tumour (11).

In addition, the recognition that many cancers are defined by common hallmarks such as abnormal metabolic function, pioneered by Hanahan and Weinberg (2011), has provided the possibility that the differences between tumour and normal cells could be defined as therapeutic targets (12). Experimentally observed metabolic differences in GBM studies are thought to be a combined result of oncogenic drivers, the tumour microenvironment (TME) and the presence of distinct cell populations such as GSCs (13, 14). Despite controversial beginnings, the acceptance of a cell type with characteristics distinct from the tumour bulk conferring resistance to standard treatment has led to a widespread belief that the eradication of GSCs would hinder tumour initiation, reestablishment and greatly improve patient outcome (15, 16). Therefore, there is an increasing aim to understand the apparent intrinsic metabolic plasticity of these cells and their ability to adapt and compensate for extreme environmental stressors such as toxic chemotherapeutics (17). Furthermore, it is becoming increasingly clear from the field of cancer research that the combination of multiple therapies may be the most promising approach to overcome heterogenic treatment responses of different cells (18, 19).

The objective of this review is to investigate experimental evidence for GSC metabolic flexibility and particularly bioenergetic capacity in comparison with normal and tumour bulk cells. Due to the pressing need for increasing GBM survival beyond such dismal figures, this review also aims to give an overview of the rationale behind new metabolic strategies (both experimental and clinical) for GBM treatment, with a focus on the GSC population. 

## GSCs and Cancer Stem-Like Cell Models

**Figure 1:**
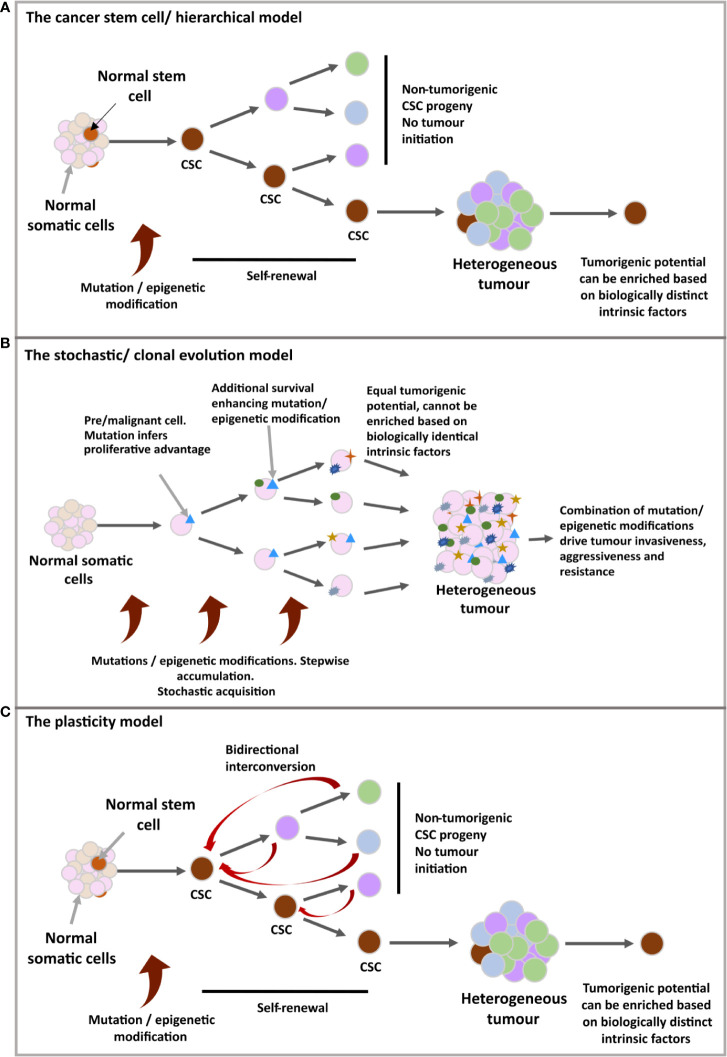
**(A)** The cancer stem cell/hierarchical model: The CSC benefits from inherent stem cell features to support malignancy; unlimited clonal expansion and self-renewal. The self-maintaining CSC population(s) are believed to differentiate in a reversible manner, producing different tumoral populations of faster proliferating cells with limited lifespan. A hierarchy is set up in which CSCs define a biologically distinct subdivision of a tumour, giving rise to further behaviourally and functionally heterogenous, non-tumorigenic cells of the tumour bulk. **(B)** The stochastic/clonal evolution model: Following neoplastic induction *via* oncogenic mutation, rapid proliferation, in combination with cumulative mutation acquisition would give rise to variants with additional selective advantages. In this model, any malignant cell is assumed as having an equal probability for tumour initiation due to identical biological features and the stochastic nature of mutation acquisition, as well as the unpredictable influence of external cues on behavioural shifts. Therefore, tumour initiating ability cannot be isolated or enriched for. **(C)** The plasticity model: A more flexible model, ‘merging’ the two previous models. Possible incorporation for bidirectional interconversion between cellular potencies, including the retrodifferentiation of non-cancer stem cells to reacquire stem characteristics.

Cancer Stem Cells (CSCs), (see **Figure 1**- stem cell/hierarchical model) in some cases are thought to derive from the mutation of non-neoplastic stem cells, recapitulating certain stem cell properties and the potential to reconstitute a tumour through unidirectional symmetric self-renewal of the CSC pool and asymmetric divisions to generate the differentiated tumour bulk (13, 20–22). However, contradictory experimental evidence has led to a contrasting model (see **Figure 1**- stochastic/clonal evolution model) in which the selective oncogenic mutation of any somatic cell could progressively accumulate mutations that produce a stem-like phenotype, forming several CSCs clones which have selective growth and evolutionary advantages over others (23–25). These linear models have since been hybridised (see **Figure 1**-plasticity model) with the suggestion that all the cells forming the tumour bulk have the potential to become CSCs through a dedifferentiation process (26, 27).

**Figure 2:**
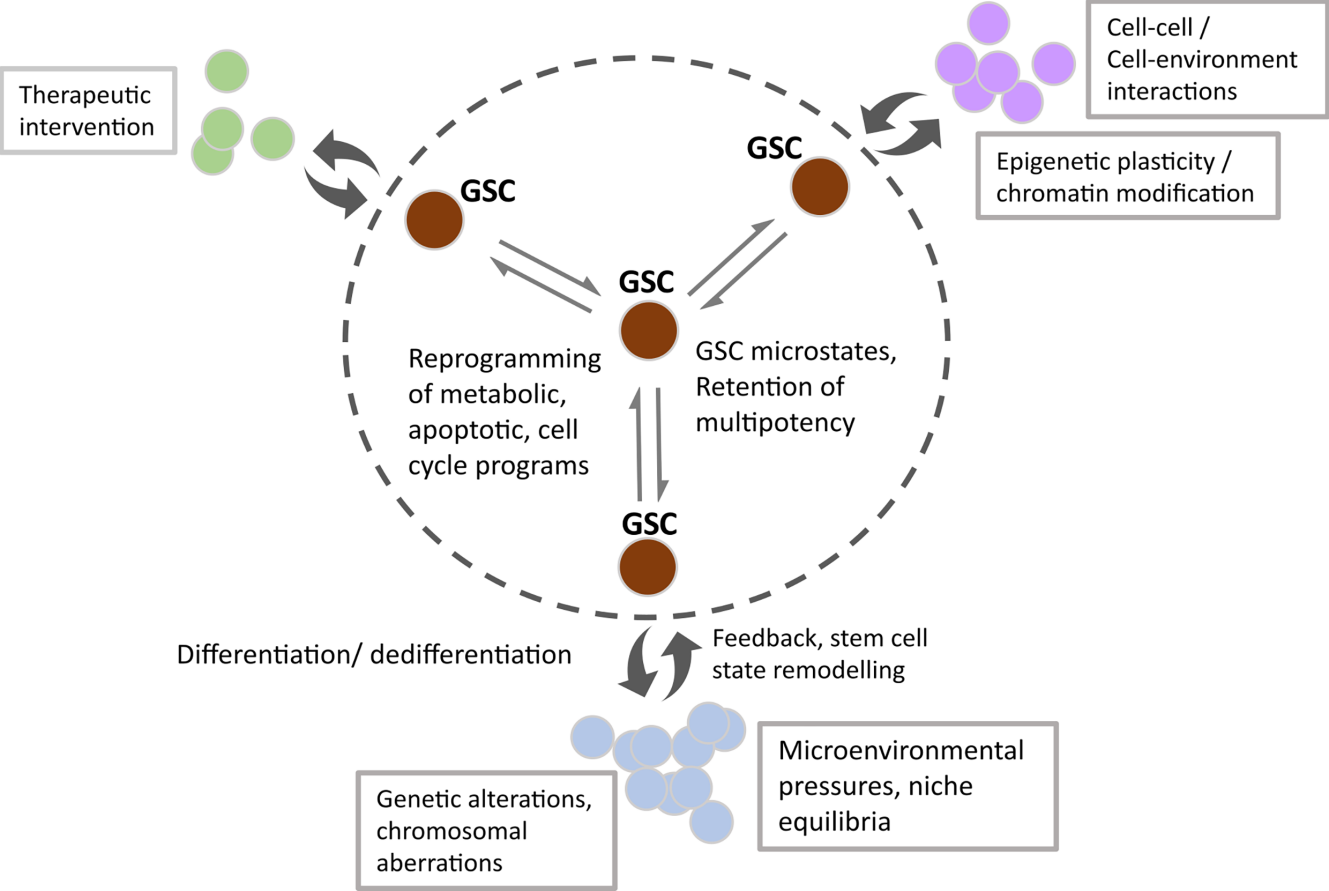
Schematic showing the microstate transitions within the GSC population (within the circled dotted line) due to enhanced transcriptional and epigenetic potential, underpinned by extrinsic cues. Double headed arrows represent dynamic transitioning between GSC states, resulting in cellular reprogramming of metabolic, apoptotic and cell cycle programs. Non-tumorigenic subpopulations emerge due to the reversible differentiation and feedback of GSC states (double-headed curly arrows). Factors affecting transition state, subpopulation size and dynamic fluxing between states include external cues such as therapeutic intervention, cell-cell/cell-environmental interactions and spatial tissue characteristics. Intrinsic changes manifest as subpopulations with distinct genetic/chromosomal aberrations and epigenetic programmes.

Singh et al. first described the concept of brain tumour initiating cells based on experimental data suggesting that only the Prominin 1/CD133+ population within GBM had the ability to initiate brain tumours in non-obese, diabetic/severe combined immunodeficiency (NOD/SCID) mice, compared with the CD133- negative population (13). Their group proposed that this glioma stem-like population may follow the unidirectional stem cell/hierarchical model of cell division described above, also reviewed by Singh et al. (13, 28). More recent evidence for this finding includes studies by Lan et al. in which lineage tracing and lentiviral DNA barcoding of NOD/SCID/IL2rγ^null^ mice implanted with GSC clones revealed the retention of a proliferative hierarchy (20). By contrast, subsequent studies have shown that the CD133-population also have brain tumour initiating properties, suggestive of potential non-hierarchical phenotypic alterations (29–32). Although the field recognises the challenge of identifying a single specific glioma stem marker (originally thought to be CD133) or combination of markers to define a specific developmental duration, it’s possible that stochastic marker expression such as CD133, CD15 and CD44 could confer a survival advantage (33, 34). In this way, the plasticity model looks to be a more accurate description of the development and behaviour of these cells, giving rise to the vast heterogeneity observed within a GBM tumour (33). Suva et al. demonstrated that neurodevelopmental transcription factors: sex determining region Y box 2 (SOX2), oligodendrocyte transcription factor (OLIG2), POU domain class 3 transcription factor 2 (POU3F2) and spalt like transcription factor 2 (SALL2) can be used to artificially reprogramme single cell primary glioblastoma cultures to a stem-like state, provides evidence for central nervous system (CNS) cell susceptibility to hierarchical reversal (35, 36). Furthermore, other groups have used mathematical modelling to predict stem cell marker combinations which may reflect plasticity during glioblastoma growth and dictate phenotypic heterogeneity within the stem cell population (14). Collectively, this evidence has led to novel interpretations of the GBM energetic landscape consisting of intrinsic diverse transcriptional and epigenetic microstates as well as plasticity in response to extrinsic cues as one of the most challenging concepts to overcome for treatment success (represented in **Figure 2**) (14).

## Altered Metabolism in GSCs

**Figure 3:**
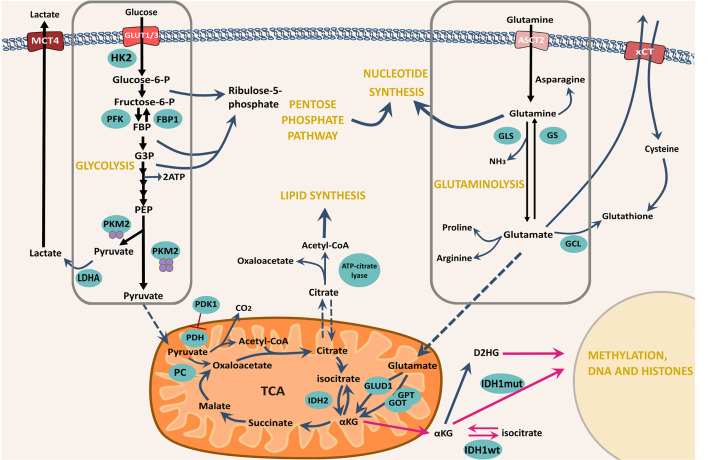
Schematic showing some of the major metabolic pathways for GSC bioenergetic function and potential adaptability. The import of both glucose and glutamine are emphasised as the major nutrients available for cellular uptake though their respective transporters. Glucose is imported through the cellularly expressed glucose transporter, in this case either GLUT1 or GLUT3 and is enzymatically processed in the cytoplasm to pyruvate. Glycolytic processing can yield intermediate precursors largely subject to processing *via* the PPP – a major nucleotide synthesis pathway. Complete glycolytic processing to pyruvate is determined by the final enzymatic step - conversion of PEP by PKM2, yielding either lactate for export through MCT4 or pyruvate for mitochondrial entrance and processing *via* TCA and OXPHOS. In addition, Glutamine is imported *via* the ASCT2 transporter and enzymatically converted to glutamate *via* GLS. The reverse reaction is catalysed by GS. Direct conversion of Glutamate to cytoplasmic glutathione can take place *via* GCL, however, indirect conversion can also take place *via* xCT export, coupled to cystine import. Glutamate can be processed further to synthesise amino acids and lipids but can similarly be used for TCA anaplerosis *via* mitochondrial import and conversion to αKG by the transaminases *GPT* and *GOT* or GLUD. Black arrows represent glycolysis and glutaminolysis. Blue curly arrows represent the shuttling of intermediates from glycolysis and glutaminolysis and their processing by subsequent enzyme-catalysed reactions. Blue dashed arrows represent mitochondrial import/export. Magenta arrows represent the reactions that take place as a result of IDH1/2 mutations. αKG, α-ketoglutarate; ECT, Electron transport chain; FBP1, Fructose-1,6 bisphosphatase 1; G3P, Glyceraldehyde-3-phosphate; GCL, Glutamate-cysteine ligase; GLS, Glutaminase; GLUD1, Glutamate dehydrogenase; GLUT1/3, Glucose transporters 1/3; GOT, Glutamic oxaloacetic transaminase; GPT, Glutamate pyruvic transaminase; GS, Glutamine synthetase; HK2, hexokinase 2; IDH, Isocitrate dehydrogenase; LDHA, lactate dehydrogenase A; MCT4, Monocarboxylate transporter 2; OXPHOS, Oxidative phosphorylation; P, Phosphate; PC, Pyruvate carboxylase; PDH, Pyruvate dehydrogenase; PDK1- Pyruvate dehydrogenase kinase 1 PEP- Phophoenolpyruvate; PFK, Phosphofructokinase; PKM2, Pyruvate kinase M2, TCA, The citric acid cycle.

Cellular metabolic reprogramming is considered a novel emerging hallmark of cancer as evidenced by Hanahan and Weinberg’s ‘Hallmarks of Cancer: The Next Generation’ (2011) (12). The identification of malignant metabolic alterations conferring advantages for cellular growth and resistance has now become a major research aim, as reviewed by Tennant et al. (37). Experimental studies evidencing GSC superior resistance against current therapeutics have led to the suggestion that inherent metabolic plasticity allows these cells to adapt and compensate, and in some cases initiate the conversion of tumour bulk cells towards a stem-like phenotype to adopt this resistance (38, 39) (See **Figure 3** for a schematic representation of GSC metabolism). Therefore, it is important to consider that GSCs may be metabolically diverse from both normal somatic cells as well as cells of the tumour bulk, that have been well studied for malignant transformation of these processes.

In normal cells when oxygen is abundant, differentiated mammalian cells fully oxidise extracellularly imported glucose in a highly efficient series of reactions. Glucose uptake is regulated through glucose transporters (GLUTs) into the cellular cytoplasm where it can then be processed to pyruvate through multiple enzyme-catalysed reactions. Pyruvate can be shuttled into the mitochondrial matrix for entrance into the tricarboxylic acid cycle (TCA) and oxidative phosphorylation (OXPHOS), yielding approximately 30/32 adenosine triphosphate (ATP) molecules for every molecule of glucose imported (40).

However, the early investigations into metabolic energy alterations in tumours by Otto Warburg and Carl and Gerty Cori in the 1920s revealed the paradoxical observation that cancer cells preferentially respire using glycolytic lactate production despite the presence of oxygen; later known as ‘The Warburg effect’ (41). This phenomenon, also referred to as the process of ‘aerobic glycolysis’ *i.e.* using anaerobic glycolysis in an oxidative environment, only yields approximately two molecules of (ATP) per glucose molecule. The finding has since precipitated widespread acceptance that increased glucose uptake is a shared cancer trait and can be exploited by positron emission tomography (PET) to inform clinical diagnosis of malignancy (42). PET measurements of glucose and oxygen processing in 14 patients with high grade tumours reported by Vlassenko et al. showed increased aerobic glycolysis that was associated with significant tumour proliferation and aggression, correlating with poor patient survival (43).

Since rapid cell division requires large concentrations of cytoplasmic macromolecular precursors for building new cells, it is believed that reducing glucose processing at pyruvate as described by Warburg, facilitates the diversion of carbon through alternative biosynthetic processes (44). The pentose phosphate pathway (PPP) is mainly responsible for nucleotide biosynthesis and rapid flux has been described as a major driver of proliferation for the ‘Warburg phenotype’ (45). In fact, PPP functioning was shown by De Preter et al. to be instrumental in a range of malignancies such as SiHa human cervix squamous cell carcinoma using pharmacological inhibition and enzymatic siRNA knock down of the pathway, resulting in a dramatic decrease in proliferation (45). A combination of studies has also been instrumental in understanding that fine tuning between glycolytic and PPP flux can lead to phenotypic balancing in GSCs, with a hypoxia driven metabolic switch to non-oxidative glucose processing causing an initial reduction in PPP enzyme expression, provoking cell migration (46, 47). Moreover, investigations of cells exposed to long-term hypoxia showed that PPP enzymes vital for proliferation can become upregulated, mirroring the phenotype of oxygenated GSCs from hypoxic culture, carried out by Kathagen et al. (46, 48). In addition to the PPP, ^13^C nuclear magnetic resonance (NMR) spectroscopic analysis of patient high grade glioma samples obtained by Maher et al. revealed additional glucose shuttling into other enzymatic reactions for cellular glutamate, glutamine and glycine pool replenishment (49).

Key regulators of the Warburg phenotype have been investigated. Pyruvate kinase (PK) - the final control point in the glycolysis pathway, exhibits pivotal roles in sensing cellular metabolic state and functioning as a rate-limiting enzyme (50). In addition, the unequal expression of isoforms (PK-M1 and PK-M2) has been described for cancers including GBM, imperative for dictating the energetic fate of glucose (51, 52). Isoform expression analysis by Mukherjee et al. revealed a much greater PK-M1 expression in the normal brain, contrasting with PK-M2 in grade I-IV astrocytoma specimens. In addition to this disparity, PK-M2 mRNA showed 3-5 times higher expression in GBM compared to grade I-III gliomas, indicating that dramatic increases in expression could enhance tumour severity (52). Constitutively active PK-M2 exists as a tetramer, favouring the production of pyruvate and TCA cycle processing for the production of ATP through OXPHOS (53). However, this enzyme is also susceptible to post-translational modification and allosteric regulation by fructose 1,6-bisphosphate (FBP) making it unstable and likely to exist as a dimer with lower affinity for phosphoenolpyruvate (PEP), promoting glycolytic intermediate accumulation (53). In fact, PKM2 has been used as a biomarker for GBM malignant growth in studies by both Witney et al. and Beinat et al. in which PET imaging with the experimental radiotracer 1-((2-fluoro- 6-[^18^F]fluorophenyl)sulfonyl)-4-((4-methoxyphenyl)sulfonyl)piperazine ([^18^F]DASA- 23) was used to assess the glycolytic response of cells to a range of current treatments (54, 55). The effectiveness of this radiotracer for diagnosis of suspected GBM cases is currently being investigated in a phase I clinical trial (trial identifier: NCT03539731).

## Oncogenic Drivers of Aerobic Glycolysis in GBM

Classically, oncogenic events have been examined for their role in the dynamic alteration of cellular metabolism and due to the ground-breaking description of the Warburg effect, the focus of many studies has been key drivers of this malignant process. There is an abundance of studies that illustrate a correlation between malignancy and increasing concentrations of glycolytic biosynthetic machinery such as GLUT1/3 and glycolytic enzymes for accelerated pathway flux (56, 57).

For GBM, common driver and tumour-suppressor genetic alterations include phosphoinisitide 3-kinase (PI3K) mutations for uncontrolled signalling, driving the continual activation of protein kinase B/Akt and leading to high rates of glucose import (58). Characteristic to GBM, upstream activation of PI3K often takes place through epidermal growth factor receptor (EGFR) *via* amplification or mutation as well as loss of PTEN antagonism (59). Crucially, PI3K/Akt signalling can be activated downstream of a wide array of growth factor receptors including platelet-derived growth factor receptor (PDGFR) – normally implicated in the mediation of tumoral proliferation predominantly in the PN GBM subtype (60). In contrast, the PDGFR has also been shown to regulate glycolysis in GSCs independently of proliferation (60). Constitutive Akt activation has been repeatedly implicated in tumoral glucose ‘addiction’, often being termed the main instigator of the aerobic switch, involved in elevated GLUT expression, membrane translocation and the regulation of carbon biosynthetic shuttling (58, 60).

In addition, changes in hexokinase (HK) expression have been reported in GBM studies such as those by Wolf et al., showing that higher GBM grades express higher levels of HK2 leading to the promotion of cell survival and growth (61). Moreover, siRNA knockdown of HK2 using intracranial xenografts conferred increased tumour invasion but less ability to proliferate and carry out angiogenesis (61). Furthermore, transcription and growth factor studies in hepatocellular carcinoma cell lines have helped delineate signalling events that can give rise to HK2 expression, including cyclic adenosine monophosphate (cAMP), glucagon, mutant p53, insulin growth factor (IGF), hypoxia inducible factor-1α (HIF-1α) during hypoxia and Myc signalling (62, 63). PI3K/Akt signalling has also been shown to stimulate HK2-mediated cell survival *via* mitochondrial translocation and interaction with voltage-dependent anion channels in HeLa cells, preventing the binding of bcl-2-like protein 4 (BAX), and increasing the release of cytochrome C (64). A depleted level of HK2 antagonism by downregulation of miR-143 in GSCs has been established to increase the self-renewal potential of these cells in studies by Zhao et al. Lentiviral miR-143 transfection of GSCs by the group showed decreased tumorigenicity even under hypoxic culture, suggesting that loss of miR-143 is instrumental for GSC progression, favourably implicating miR-134 upregulation as a therapeutic target (65).

c-Myc has also been frequently described as another major driver of aerobic glycolysis, with overexpression causing the downstream upregulation of HK2, PKM2 and lactate dehydrogenase A (LDHA) in studies by Tateishi et al. (66). The mechanistic target of rapamycin complex 2 (mTORC2), a downstream nutrient sensor of Akt involved in the control of lipid and protein synthesis has also been shown to activate Myc in the absence of upstream Akt (67). Furthermore, the retrospective analysis of patients with brain metastases by Neider et al. showed elevated LDH levels, with additional studies in GBM showing that tumour derived LDH5 can confer immune escape by impeding the recognition of the tumour by natural killer cells (68, 69). Additionally, higher LDH-A expression in studies by Kim et al. using U87 GBM cells was shown to be associated with faster tumour growth kinetics, mirroring that of astrocytes (70). Moreover, the small molecule inhibition of LDH-A by Daniele et al. promoted cellular apoptosis in U87 cells as well as an induction of GSC differentiation of neurospheres (71).

Lastly, inherently characteristic to GBM categorisation, mutant IDH1/2 has a significant impact on tumoural prognosis (72). Wild type IDH catalyses the recognised TCA conversion of isocitrate to α-ketoglutarate (α-KG) and is predominantly associated with primary/*de novo* cases of GBM (73). α-KG can function as a co-factor for enzymes such as dioxygenases and histone demethylases involved in epigenetic modification (74). However, mutant IDH has been observed in more than 90% of secondary GBM cases, catalysing the conversion of isocitrate to the clinically recognised oncometabolite D-2-hydroxyglutarate (D-2-HG) (73, 75). D-2-HG is involved in the competitive inhibition of the α-KG dependent dioxygenases, leading to alterations in global deoxyribonucleic acid (DNA) hypermethylation [including O^6^-methylguanine-DNA methyltransferase (*MGMT*) promoter methylation] and differentiation suppression (74, 76). In addition, IDH1 mutant glioma is associated with the CpG island methylator phenotype (CIMP) first described for colorectal cancer, and in GBM (G-CIMP) is associated with the PN molecular subtype (77–79). This mutation is clinically associated with a higher frequency of occurrence in younger patients and largely correlates with longer overall survival time (7, 78, 80).

## GSC Metabolic Resilience

### Glucose Oxidation

In one of the first assessments of CSC metabolic states, Vlashi et al. studied the oxygen consumption rate and external acidification rate of GSC neurospheres derived from three independent GBM samples, compared with differentiated progenies cultured as monolayers (81). The group found that GSCs exhibited lower glucose uptake rates, lower lactate production, higher ATP levels and were therefore less glycolytic. Moreover, the group proposed that differentiation could induce a switch from a dominant use of OXPHOS for ATP production to glycolytic dependency (81). By contrast, GSCs derived in the same way from U87 tumours by Zhou et al. showed no significant difference in ATP levels in hypoxic versus normoxic conditions (82). When assessed under exclusive normoxia, elevated GSC lactate production was observed as well as a 4-fold increase in glucose uptake, suggestive of predominant glycolysis (82). Rationalisation of this result with findings from other GSC studies include the increased expression of neuronal GLUT3 by these cells for higher affinity glucose uptake, with GLUT3 knockdown in studies by Flavahan et al. conferring a significant decrease in the growth of GSCs (83). In addition, Zhou et al. showed that GSCs derived from two GBM surgical specimens expressed lower levels of voltage-dependent anion channel 2 (VDAC2) than the differentiated phenotype, believed to be essential for preservation of stem cell features, tumorigenicity and phosphofructokinase (PFKP) mediated glycolysis (84).

By collectively interpreting contrasting results from the GSC studies discussed, more detailed analysis reveals additional complexity, suggestive of heterogenous metabolic phenotypes within a single tumour (85). For example, Hoang-Minh et al.’s separation using CellTrace dyes and metabolic characterisation of GBM cell cycling speeds relative to bioenergetic strategy indicated that slow cycling cell (SCC) tumour populations, believed to be enriched in GSCs, were shown to survive predominantly using OXPHOS and lipid metabolism (86). SCCs derived from primary human GBM cell lines also displayed elevated chemoresistance and invasive capacity (86). Furthermore, a multitude of studies have recorded GSC metabolic subtype disparity, in which Gene Set Variation Analysis (GSVA) of TCGA GBM subtypes reveal that MES GBMs predominantly correlate with glycolytic pathways (87). Moreover, the metabolic enzyme analyses of MES GBM have revealed preferential glycolytic enzyme expression such as aldehyde dehydrogenase 1A3 (ALDH1A3) (87). This metabolic heterogeneity has been further highlighted by Duraj et al.’s application of 4 different metabolic drugs (metformin (MF), dichloroacetate, sodium oxamate and diazo-5-oxo-L-norleucine) to three GSC types, shown to exhibit different drug sensitivities due to initial cellular glycolytic/oxidative tendencies (88).

The employment of updated techniques to directly quantify metabolites for metabolic flux analysis *in vitro* and *in vivo* has also catalysed an increased acceptance for metabolic plasticity, challenging the categorisation of discrete malignant metabolic phenotypes pioneered by Warburg  (89). In more contemporary studies, Shibao et al. derived isogenic glioma initiating cells (GICs) from neural stem cells (NSCs) expressing the H-Ras^V12^ oncoprotein and showed that orthotopic primary tumour initiation was independent of initial cellular metabolic state and glycolytic enzyme expression (90). Furthermore, clonally derived GSCs from the same GIC 14 days post *in vivo* initiation showed tumour sustenance of metabolic diversity, thought to demonstrate the possibility for coexistence of GSCs with different bioenergetic strategies within the same tumour, relative to their environmental niche (90). Transcriptional up/downregulation of the glycolytic enzymes HK2, PKM2, LDHA and PDK1 were also recorded following the observation that cells were able to glycolytically compensate on exposure to hypoxia, and vice versa using OXPHOS after glycolytic inhibition, indicative of cellular metabolic coping mechanisms for continued biosynthesis (90). 

### Glutamate Metabolism

Regardless of the potential that the Warburg hypothesis brought for glycolytic inhibitor targeting, cancer cell survival and resistance remains a barrier to therapeutic success. As time has passed, the elucidation of a tangled cellular metabolic network has precipitated increasing futility of unimodal targeting strategies excluding tumoral heterogeneity and TME remodelling. Therefore, it is important to consider how the glycolytic pathway fits into a larger picture of cellular metabolism (**Figure 3**) and thus, isolated targeting of this pathway may be increasingly misjudging the tangled network of cell coping mechanisms. DeBerardinis et al. noted that simply observing a heavy cellular reliance on aerobic glycolysis may be accessory to a temporary metabolic strategy, facilitating the production of proliferative precursors as opposed to a complete impairment of the oxidative pathway (91). The group also illustrated that the catabolism of glutamine in addition to other carbon sources such as acetate shown by Mashimo et al. may be an imperative feature of Warburg’s observed cancer cell phenotype, ‘picking up’ the burden of TCA anaplerosis and nicotinamide adenine dinucleotide phosphate (NADPH) synthesis for continued cell maintenance (91, 92).

Glutamine is an abundant plasma nutrient essential for cellular catabolic regulation, flux and processing of carbon, nitrogen and reducing equivalents (91). Integrating results from the study of GBM, other cancers and neurodegenerative disorders, a crucial balance between glutamine synthesis and catabolism has been recognised (93). Glutamine uptake by the alanine/serine/cysteine transporter 2 (ASCT2) transporter is followed by cytoplasmic glutaminase (GLS) conversion to glutamate and subsequent fate processing dependent upon cell requirements. Glutamate can be directly processed to the antioxidant glutathione *via* glutamate-cysteine ligase (GCL) enzymatic combination with intracellular cysteine or exported though through xCT (cysteine/glutamate transporter) subject to cysteine import (94). The GBM xCT mediated export of glutamate has been shown to confer tumoural invasive expansion of rat striata implanted glioma cell clones with staining showing neuronal degeneration and inflammation of the surrounding TME (95). Moreover, immunoprecipitation analysis of three glioma cell lines and two primary human GBM cells by Tsuchihashi et al., revealed the control of xCT surface expression was subject to direct interaction with the EGFR intracellular domain, a mechanism which has since been further delineated by the group  (96, 97). Glutamate is also instrumental for non-essential amino acid synthesis as well as purine and pyrimidine nucleotide precursors (94). Furthermore, glutaminolysis for the continued TCA flux and maintenance of biosynthetic intermediates has been a prominent feature in the study of cancer transformation.

In the healthy brain glutamine production *via* the glutamine synthetase (GS) catalytic condensation of glutamate and ammonia exclusively by astrocytes can be cooperatively utilised by neuronal GLS for glutamate hydrolysis and subsequent neurotransmission; termed ‘the glutamine-glutamate cycle’ (98). The ammonia utilised by GS is a product of amino acid breakdown and this enzyme is therefore essential for detoxification as well as uncontrolled neurotransmission through clearing glutamate from the synaptic cleft. However, importantly astrocytic *de novo* glutamate synthesis for neurotransmitter pool maintenance is dependent upon the enzyme pyruvate carboxylase (PC) and has been used as a specific marker of astrocytes. PC shuttling of glucose-derived pyruvate into the TCA for glutamate synthesis is subsequently converted to glutamine through GS.

An unmistakable metabolic adaptation drawn from glioma studies including GBM is an ‘addiction’ to glutamine for survival and growth, particularly in hostile conditions. *In vitro* experiments by Wise et al. using the paediatric GBM cell line SF188 revealed glutamine growth dependence, such that removal of glutamine from the culture medium inhibited cell survival despite the presence of glucose (99). This finding is well established for other cells including cancer with many early studies reporting cell survival in glutamine and nucleoside supplemented culture media in the absence of any sugar (100–102). Furthermore, inhibition of the established oncogenic drivers of glucose addiction PI3K and Akt by Wise et al. showed no effect on glutaminolysis mediation, precipitating the finding that elevated *MYC* expression resulted in upregulation of glutamine dependence genes (99). Myc was shown to stimulate increased *SLC1A5* (ASCT2) transcription for higher rates of uptake and glutamine mediated TCA anaplerosis, essential for the replenishment of precursors for growth limiting macromolecule synthesis (99). This data implied the possibility that anaplerotic nutrient use was driven by discrete oncogenic systems. Other studies to further analyse high *MYC* expression and nutrient dependence in a range of human cell lines including human lung fibroblasts and human lymphoblastoid cells have found that Myc dependent miR-23a and miR-23b transcriptional repression upregulates GLS expression as well as sensitising cells to apoptosis during glutamine deprivation (103, 104). 

### Anaplerotic Balancing

Besides studies implicating glutamine as an extremely important molecule for TCA anaplerosis and cell survival, it is important to recognise that a balance exists between glucose, glutamine, and other cellular respiratory substrates. As mentioned for astrocytic systems, glucose can be catabolised to pyruvate and diverted away from PDH mediated entrance into the TCA, instead being carboxylated to oxaloacetate by PC. Nonetheless, observations consistent with the Warburg effect have rarely implicated glucose as the predominant precursor for neoplasm anaplerosis, supported by findings from metabolic flux experiments in well established “glutamine addicted” SF188 paediatric GBM cells with negligible PC activity (91). In fact, *in vitro* SF188 metabolic flux analysis has shown near exclusive nutrient favouring, with DeBerardinis et al. stating that cells derive up to 90% of anaplerotic oxaloacetate production from glutamine (91). Nonetheless, studies in human lung cancer tissue and hepatocellular carcinoma cell lines *in vivo* and *in vitro* respectively have evidenced preferential PC anaplerosis mediation, resulting in different sensitivities to experimental nutrient withdrawal (105, 106). As a result, some GBM experimental designs have since focussed on delineating neoplasm anaplerotic carbon source flexibility during nutrient deprivation. This is of clinical importance due to the potential for therapeutic confounding resulting from cellular complementation and survival mechanisms using alternative pathways (105). Accordingly, further analysis using GLS suppression in SF188 and adult LN229 GBM cell lines by Cheng et al. have since revealed that despite limited growth, cells are able to compensate using other GLS independent amidotransferase-catalysed glutamate generating pathways as well as glucose-derived carbon incorporation into TCA intermediates through PC (105). The additionally observed upregulation of PC after glutamine deprivation led the group to conclude: “PC is dispensable for growth of glutamine-replete glioblastoma cells, but required when glutamine supply is limited’’ (105).

Further to studies using single cell lines, the use of patient derived primary GBM samples has since been imperative for the delineation of tumoural metabolic heterogeneity. Metabolic analysis of 14 patient GBMs by Oizel et al. revealed phenotypic disparity and clustering as two distinct groups (107). One group exhibited substrate flexibility including glutamine utilisation for TCA directed nicotinamide adenine dinucleotide (NADH) formation, as well as higher expression of *SLC1A5*, *GLS* and Glutamic-Oxaloacetic Transaminase 1 (*GOT*) (107). The other exhibited glucose dependency for survival and growth. Substrate removal and substitution experiments revealed that the metabolically flexible phenotype was essential for maintaining cell proliferation when glucose was removed (107). In addition, the long-term inhibition of glutamine metabolism using the inhibitor, epigallocatechin gallate allowed cells to use glucose mediated TCA flux (107). Data such as these have been essential for recognising the concurrent existence of cells with different substrate sensitivities within a whole tumour. Later, the group combined metabolic analysis with molecular subtyping data, revealing that cells with higher substrate flexibility and metabolic adaptation potential could be categorised as MES, whilst glucose dependent cells represented one of the other subtypes. Further extension of experiments to the U87 cell line associated with GSC features, conformed to the MES subtype and thus, exhibited glutamine-mediated anaplerotic shuttling (107). The MES subtype has been heavily implicated for hypoxic survival and represents a subset of GBM (9, 10). These findings correlate with experimental data from Flavahan et al. using patient derived GSCs confirmed by assessing self-renewal, proliferation and expression of stem cell markers to show the association of high GLUT3 expression with GSCs in the PN subtype (83). However, earlier isotopic analysis studies using ^13^C mass spectrometry of isolated GSCs in neurospheres cultured from GBM orthotopic mouse models by Marin-Valencia et al. displayed rapid consumption of glucose for PC mediated anaplerosis and mitochondrial oxidation in addition to *de novo* glutamate production (108). Corresponding transcription data revealed the low expression of GLS in contrast to PC and GS levels, accessory to the observation that glucose-derived glutamate was used to maintain a large intracellular pool of glutamine, mediated by GS (108). Corroborating data from other studies have shown that GBM cells with high GS expression showed little to no reliance on extracellular glutamine uptake for growth preservation *via* glutaminolysis, but instead a dependence on *de novo* glutamine synthesis for intracellular pool replenishment (109). Assumptions based on this data have since exclusively defined GSCs as displaying high GS levels, contrary to data from Oizel at al. in which molecular subtyping is believed to be the more dominant driver of metabolic phenotype, with both GS-positive and GS-negative GSCs described (107, 109) Molecular subtyping of the GSCs studied by Marian-Valencia et al. would have revealed whether this data correlated with finding by Oizel et al., such that these cells showed subtyping distinct from the MES phenotype.

Nonetheless, combining these study outcomes, a distinction has been made between GBM cells displaying low GS levels as being mainly reliant on glutamine for TCA anaplerosis, with the ability to switch to largely glucose as a main substrate under glutamine repression (107). In contrast, GBM cells with high GS levels frequently defined as GSCs are believed to rely upon PC-mediated anaplerosis as a major source of glutamate GS mediated glutamine pool replenishment. In this way, these cells show major similarities to untransformed astrocytic systems in which PC and GS activities are required for glucose-derived glutamine pool maintenance, with little reliance on external glutamine uptake for cell proliferation. In studies by Tardito et al. proportionate protein levels were recorded for astrocytes and GS-positive GBM cells (109). The heavy reliance on *de novo* glutamate production as observed in GSCs with high GS expression, is believed to be an abundant source of nucleotide precursors for sustained purine biosynthesis (49, 108, 109).

### GSCs as Astrocytic ‘Parasites’/Metabolic Adaptability and the TME

Metabolic crosstalk between GBM cells and the stroma in the context of plastic substrate utilisation is essential for transformed cell survival during chaotic TME dynamics. In addition, studies evidencing the ability of subpopulations of tumour cells to adapt during nutrient restriction has led to investigations of cancer-stromal alliances in a range of cancers for survival (110–112). Tardito et al. reported low kinetic uptake of glutamine from the blood by GS-negative GBM cells and the healthy brain (109). Since GS-negative GBM cells have shown high glutamine uptake for anaplerotic pathways, orthotopic mouse models of GS-negative GBM revealed that other GS expressing tumour cells as well as astrocytes were a major source of the amino acid. Moreover, co-culture of astrocytes and GS negative LN18 GBM cells revealed growth sustenance based on astrocytic glutamine production in the absence of media supplementation (109). This data shows that subject to transformation, glutamine excreted into the TME for endogenous neuronal transmission, can instead support the growth of malignant cells that require a supply of metabolic nutrients. Furthermore, Kallenberg et al.’s magnetic resonance spectroscopy data illustrated a higher concentration of glutamine in the hemispheres of GBM patients relative to healthy controls, reported as a marker for early tumour infiltration (113). In addition, cross section staining for astrocytes and GS level of human derived GBM xenografts display a symbiotic positioning of astrocytes surrounding GS-negative GBM cells (109).

## Clinical Relevance – Metabolic Targeting

### Glycolysis Inhibition – Targeting the Warburg Phenotype

The widespread acceptance of the Warburg effect as well as experimental evidence supporting this metabolic shift in GBM tumours has been instrumental for the emergence of glycolytic targeting strategies. Glucose uptake inhibition has been dominated by the use of GLUT1 antagonists such as indinavir and ritonavir, shown *in vitro* to reduce GBM cell proliferation including in GSC cell lines (114). However, despite the synergistic effect of ritonavir when combined with chemotherapeutics *in vitro*, both compounds are unable to effectively cross the blood brain barrier (BBB), and therefore not able to reach effective concentrations in the brain (114). Alternatives have included compounds such as 2-fluoro-6-(*m*-hydroxybenzoyloxy) phenyl *m*-hydroxybenzoate (WZB117), displaying successful *in vitro* inhibition of GSC self-renewal ability (115). However, despite the beneficial effects observed for the inhibition of this transporter, the robustness of the targeting strategy has been challenged due to widespread GLUT1 expression in the human brain and therefore potential for off target effects (116). Consequently, GLUT3 has since emerged as a more promising target with protein expression analysis by Flavahan et al. showing elevated levels of the transporter in GSCs (83). Moreover, studies by Cosset et al. have interrogated GLUT3 expression further, showing that targeting with therapeutics may specifically inhibit classical/proneural cells within GBM tumours (117).

Other glycolytic inhibition strategies have included the suppression of HK2 with antifungals, as well as use of the glucose analogue 2-deoxy-D-glucose (2-DG) for competitive inhibition of cellular glucose uptake, and overall reduction in glycolysis (118, 119). The depletion in ATP production resulting from 2-DG treatment has been shown to promote cellular endoplasmic reticulum (ER) stress, followed by the unfolded protein response (UPR), similarly observed as an effect post radiotherapy (119). It is believed that GSCs have a greater ability to escape this stress with superior autophagic pathway promotion and reestablishment of ER homeostasis (120). Therefore, currently, there are efforts to promote tumour apoptosis by preventing autophagic and UPR mediated ER homeostasis reestablishment, with some groups finding that blocking ER stress altogether and the downstream protective mechanisms could enhance the cytotoxic effects of small therapeutic compounds such as shikonin (121). An increasingly established downstream effector of the UPR is 78-kDa glucose-regulated protein (GRP78) - a member of the heat shock protein family, instrumental for downstream activation of ER homeostasis regulators as well as observed cell surface translocation, characteristic of invasive cancers (119, 122). Whilst the translocation of GRP78 from the ER to the cell surface is not well understood, it is believed that GRP78 may also regulate cellular interactions with the surrounding TME (123).

### The Ketogenic Diet

Further to treatment strategies requiring administration of small molecule inhibitors, the modification of patient dietary nutrient intake has become a potentially effective approach for diverting tumour metabolism, such as short-term starvation shown by *in vitro* colon cancer studies for apoptosis induction (124). The dietary reduction of serum glucose concentrations involves restricting carbohydrate consumption, traditionally implemented by following a 4:1 fat to protein and carbohydrate ratio (commercially available as KetoCal^®^) that is well tolerated in patient studies for targeting GBM energy metabolism (125–127). As a result, multiple clinical trials are either complete or ongoing evaluating the safety/tolerance and effectiveness of the ketogenic diet (KD) as an adjuvant to current treatment in GBM: NCT01865162, NCT023939378, NCT04691960, NCT02302235, NCT00575146, NCT03451799, NCT01754350, NCT03075514, NCT03278249, NCT01535911, NCT04730869, NCT02286167. This dietary limitation forces the liver to metabolically adapt, using fats to produce ketone bodies which can be used as an alternative fuel source for cellular energy metabolism; originally pioneered for the beneficial reduction in seizures in epileptic children (128). Furthermore, Otto Warburg’s description of tumoural exploitation of aerobic glycolysis provided initial rationale for the approach in GBM, suggested as an effective strategy to slow tumour growth and reduce ability of cells to exhibit a Warburg phenotype (129). As such, the efficacy of this treatment has been shown by Abdelwahab et al’s. use of a bioluminescent mouse model of malignant glioma to confer increased survival (127). Moreover, the retrospective statistical analysis of patient data suggests that postoperative hyperglycaemia is associated with poor survival; an effect that could be suppressed by lowering glucose levels during treatment (130–132).

Despite studies demonstrating efficacy of the KD for increased GBM survival, Sperry et al. have explored GBM intratumoural heterogeneity to reveal that populations of tumour cells could in fact thrive using ketone bodies and upregulated fatty acid oxidation for survival, therefore mitigating the effectiveness of the KD (133). However, the authors highlight that the KD in combination with additional metabolic inhibition of enzymes such as carnitine palmitoyltransferase 1A (CPT1A) – the rate-limiting enzyme for fatty acid oxidation, or implementation of additional calorie restriction to exacerbate the effect of serum glucose reduction could increase the efficacy of the KD, as evidenced by Shelton et al. in GBM (133, 134). Tumour survival *via* the exploitation of pathways that circumvent low glucose concentrations such as glutaminolysis have also become a concern for implementation of the KD as an effective treatment (135). Moreover, despite the maintenance of low glutamine levels in the healthy brain *via* the glutamine-glutamate cycle, studies by Takano et al. have shown that glioma glutamate secretion into the surrounding brain could be readily recycled by tumours for sustained energy metabolism and growth (95). Therefore, strategies implementing the calorie restricted KD in combination with 6-diazo-5-oxo-L-norleucine (DON) – a gluatminolysis antagonist has been tested *in vivo* GBM mouse models by Mukherjee et al. conferring increased survival and tumour cell apoptosis (135).

Besides a reduction in glycolytic flux resulting from the KD, other downstream cellular responses have been investigated by Ji et al. using GSCs derived from both patients and cell lines (136). Culture medium high in β-hydroxybutyrate for mimicking the effects of the KD resulted in reduced GSC proliferation and tumorigenicity as well as revealing the possibility for the induction of damaging morphological and functional mitochondrial alterations (136). In addition, studies by Seyfried et al. using *in vivo* astrocytoma models exposed to restricted standard and KDs showed alterations in metabolic modulators such as a reduction of IGF-I (137). Both IGF-I, the IGF1 receptor (IGF-1R) and associated signalling network have been linked with tumour survival in a range of cancers, with experimental data showing the presence of the receptor providing protection from apoptosis following cytotoxic treatment (138, 139). Moreover, the IGFR1 exhibits elevated expression in GBM cells when compared with normal brain cells and is therefore believed to be a marker of reduced patient survival and inhibition of tumour cell apoptosis (140, 141). In fact, Zhang et al. carried out *in vitro* culture studies comparing control U87 GBM cells to those overexpressing the IGF-R1, revealing that in response to hydrogen peroxide exposure, the cells with higher IGF-R1 could inhibit apoptosis. In addition, increasing concentrations of IGF1 or overexpression of the IGF-1R resulted in Akt phosphorylation, believed to increase PI3K/Akt pathway activation and subsequent apoptosis suppression (140).

### Peroxisome Proliferator-Activated Receptors and Differentiation Induction

The 2010 retrospective review of high-grade glioma patients by Grommes et al. exposed that diabetic patients receiving treatment with PPARγ agonists (Thiazolidinediones) had a median survival increase of 13 months in comparison to those not taking this medication (142). Despite analysis of the data defining this result as not significant due to small sample size, expression data showing elevated levels of PPARγ in malignancies such as colon, duodenal, lung, prostate, thyroid, primary and metastatic breast cancer has provoked an increased interest in the clinical relevance of this receptor family, of which there are 3 mammalian members: PPARα, PPARβ/δ and PPARγ (143–148). The PPARs are part of the nuclear receptor superfamily and function as ligand-inducible transcription factors which under normal conditions bind dietary fats as well as regulating both adipocyte and macrophage biology (149).

PPAR agonists have therefore gained traction with studies by Keshamouni et al. demonstrated that treatment of non-small cell lung cancer (NSCLC) patient tumours with the PPARγ ligand troglitazone conferred anti-proliferative effects due to G0/G1 cell cycle arrest and reduction in cyclin D/E expression (145). The same cell cycle arrest has also been recorded in GBM tissue samples using the PPARγ ligand pioglitazone by Zang et al., with other research groups showing that this ligand could additionally stimulate β-catenin mediated apoptosis of GBM cell lines (150, 151). Interestingly, Chearwae and Bright (152) treated neurospheres generated from commercial GBM cell lines with the PPARγ agonists 15-deoxy-Δ^12,14^-prostaglandin J_2_ (15d-PGJ2) or all-*trans* retinoic acid to induce apoptosis and inhibit neurosphere formation and expansion through Tyk2-Stat3 inhibition (152). The notion that these ligands exert effects though the Janus kinase-signal transducer and activator of transcription (JAK-STAT) pathway has been complemented by studies by Mo et al. using PPARγ ligands in mouse embryonic stem cells (153). Since STAT3 is crucial for the self-renewal of GSCs, the potential downstream inhibition of this pathway could be important for sensitisation to chemotherapeutics such as temozolomide (TMZ), as shown by Villalva et al. (154).

Further to the delineation of different populations of GBM cells with distinct cycling kinetics by Hoang-Minh et al., enhanced cytotoxic resistance of slow-cycling populations have been described by studies performed in colon and breast tumour cells by Moore et al. despite being successful against the more proliferative tumour bulk (86, 155). Therefore, the induction of differentiation in stem populations has become a promising therapeutic strategy for cellular sensitisation to standard chemo and radiotherapy for GBM (Stupp protocol) (4). Pestereva et al. used imatinib to inhibit the PDGFR and stem cell factor receptor (c-Kit), with encouraging reductions of stem cells markers and tumorigenicity of tissue derived GSCs (156). There is also promise that PPARγ agonists could be useful for inducing differentiation of GSCs within tumours due to indications that ciglitazone and 15d-PGJ2 caused altered expression of stemness genes such as *SOX2* and *NANOG* (157). This was again tested in mouse derived NSCs cultured as neurospheres by Kanakasabai et al. with stem cell gene expression analysis before and after treatment with iglitazone or 15d-PGJ2 revealing a downregulation of stem and differentiation associated genes (158). However, despite the potential benefits of this approach, studies by Caren et al. have highlighted the importance of ensuring terminal GSC differentiation in a stable manner, due to the variable responses of cells to bone morphogenic protein (BMP) therapies. Therefore, studies are required to investigate the mechanisms used by cells to evade patterns of commitment, making them vulnerable to dedifferentiation (159).

In addition to the potential beneficial effect of PPARγ agonists on GBM, the elevated expression of PPARα recorded in GBM tissue by Haynes et al. has been complemented by data demonstrating tumour growth suppression following the use of agonists such as gemfibrozil (160, 161). This beneficial effect has also been observed in NSCLC cells, where induction of PPARα inhibited growth and angiogenesis, as well as leading to apoptosis in ovarian cancer cells when combined with PPARγ induction (162, 163). However, a compelling aspect of PPARα is that it is imperative for ketogenesis due to transcriptionally regulating 3-hydroxy-3-methylglutaryl-CoA (HMGCS) – the rate-limiting enzyme for the conversion of acetyl-CoA to β-hydroxybutyrate and acetoacetate (164, 165). Therefore, PPARα can also downregulate IGF/Akt signalling discussed for the KD as well as GLUT1 and 4 receptors, essential for supressing the Warburg phenotype (166–168).

### Mitochondrial Targeting

Further to studies revealing that GBMs undergo glucose oxidation, the discovery of mitochondrial aberrations including electron transport chain (ETC) components and mitochondrial reserve capacity have provided insight into additional GBM survival mechanisms (169). Mitochondrial DNA (mtDNA) profiling has revealed mutations in complex I, III and IV of the ETC, affecting the balance between OXPHOS and aerobic glycolysis (170, 171). GBM patient tumour tissue analysis by Lloyd et al. revealed that at least one mitochondrial DNA (mtDNA) mutation is present in 43% of patients (171). Further to this, large scale mtDNA alterations, ETC remodelling, and enzyme activity modulation has been shown to be critical for TMZ resistance in both cell lines and human GBM specimens, with chemoresistance largely driven by cytochrome C oxidase (COX) (172). Moreover, subject to research by Oliva et al., COX subunit-IV (COX-IV) was shown mainly to be associated with COX isoform I (COX-IV-I) for cellular nutrient sensing and modulation of energy production in TMZ resistant cells (172). This finding was later extended to GSCs, with COX-IV-I displaying cooperation with downstream targets to enhance tumorigenicity and self-renewal (173). Consequently, ETC pharmacological and genomic intervention has been further investigated, with results suggesting TMZ sensitisation following COX-IV-I targeting (174, 175).

Furthermore, the anti-diabetic biguanide drug, MF, has been analysed as a promising therapeutic strategy for decreasing cancer proliferation and inducing apoptosis subject to nutrient availability. Investigations in colon cancer cells have revealed that MF reversibly inhibits the function of ETC complex I subject to the presence of membrane potential and glucose (176, 177). Other studies have revealed that in the absence of glucose, MF can induce cellular apoptosis as well as a possible selective effect on CSCs (177–181). Further to this, phenformin - a MF analogue with elevated potency, was used by Jiang et al. *in vitro* and for *in vivo* tumour mouse models, revealing multiple beneficial effects including a reduction in GSC self-renewal and overall prolonged mouse survival (182). However, since biguanide use is associated with lactic acidosis induction, combining MF with the inhibition of PDK by dichloroacetate (DCA) by Haugrud et al. conferred a cell survival advantage, both reducing lactate production and inhibition of oxidative metabolism in breast cancer cells (182, 183). GBM oxidative stress induction has also been described for other compounds such as the anthelmintic drug Ivermectin, similarly shown to induce apoptosis *via* ETC complex I inhibition as well as targeting angiogenesis and the Akt/mTOR pathway in GSC cell lines (184). Interestingly, this drug also induces apoptosis in human microvascular endothelial cells (HBMEC), thus breaking an important protective and synergistic relationship believed to be critical for GBM niche maintenance (184).

### Combination Strategies

Further to the identification of isolated metabolic targets, the combination of existing GBM chemoradiation protocols with agonists/antagonists of multiple signalling pathways such as sonic hedgehog (Shh), murine double minute 2 (MDM2), p53, PI3K/mTOR, EGFRvIII, poly(ADP-ribose) polymerase 1 (*PARP1*), cyclin-dependent kinase 4/6 (CDK4/6) have exhibited cytotoxic sensitisation efficacy of GBM models (185–191). Further experimental combinatorial strategies include the use of impridone compounds for inhibiting both glycolysis and OXPHOS *via* Akt/ERK dual inhibition, c-Myc degradation and apoptosis in GSCs (192). In addition, Yuan et al. effectively used the glycolytic inhibitor 3-bromo-2-oxopropionate-1-propyl ester (3-BrOP) and the alkylating chemotherapeutic carmustine/BCNU in GSCs that were initially highly glycolytic, causing major ATP depletion and abrogation of DNA repair capacity (193). Furthermore, the development of the arsenic-based mitochondrial toxin, 4-(N-(S-penicillaminylacetyl)amino) phenylarsonous acid (PENAO) by Shen et al. to trigger mitochondrial apoptosis had better efficacy when used with DCA for dual targeting of glucose metabolism (194). Other combinations of drugs undergoing clinical development for GBM have also been tested with existing compounds such as Navitoclax/ABT-263 (a Bcl-2/Bcl-xL inhibitor) with encouraging *in vivo* low toxicity and suppression of tumour growth (195). Moreover, studies using chemotherapeutics with both MF and phenformin have shown beneficial effects tumour growth inhibition, with Jiang et al. using phenformin and TMZ *in vivo* for prolonged mouse survival (182).

However, the epidemiological identification of existing drugs with anti-tumour effects is not restricted to the biguanides, many repurposed compounds are currently under scrutiny in clinical trials in combination with existing chemoradiation protocols (see **Table 1**). Moreover, clinical trials to test the coordinated blocking of multiple different cell survival pathways has become an attractive strategy, with efforts to use multiple repurposed compounds that will work synergistically for the greatest therapeutic benefit. In 2016, the CUSP9v3 trial (identifier: NCT027703780) started, combining TMZ with Aprepitant, Minocycline, Disulfiram, Celecoxib, Sertraline, Captopril, Itraconazole, Ritonavir and Auranofin in additive treatment cycles for the treatment of recurrent GBM cases; a strategy originally proposed in 2013 by Kast et al. (216, 217). Although the results have not been released at the time of writing this review, experimental studies to test the robustness of the CUSP9 strategy have been published, including Skaga et al.’s investigations using 15 GSC cultures, derived from 15 patient GBMs including relapsed tumours (18). The group found that the combination of drugs was significantly more effective than when used alone, as well as increasing the therapeutic benefit of TMZ for sphere eradication in most of the cell lines, with the highest resistance in a proneural population (18). In addition to testing compound efficacy with conventional therapy, it has come to light that patient tumour profiling prior to therapeutic application may give a greater therapeutic benefit. Recently, the antiproliferative effects of four different compounds: MF, dichloroacetate (DCA), sodium oxamate (SOD) and diazo-5-oxo-L-norleucine (DON) were tested on 4 GSC subpopulations with different initial oxidative/glycolytic metabolism tendencies by Duraj et al. (88). The group found that GSC inhibitor sensitivity differed relative to Warburg-like/OXPHOS phenotype, and Seahorse XF to determine glycolytic/mitochondrial ATP production shifts following treatment. The authors suggest that predictive metabolic shifts despite initial bioenergetic plasticity following treatment, could be used as “metabolic priming” (88). In this way, the malignant cells could be pushed towards exhibiting a phenotype with enhanced sensitivity to subsequent small molecular inhibitors, chemo- or radiotherapy.

**Table 1:** Repurposed drugs currently in clinical trials for the assessment of efficacy against GBM.

Repurposed drug	Mechanism of action	Indication of therapeutic benefit for GSCs	References	Trial identifier	Therapeutic combination	Tumour inclusion criteria	Reported results
Metformin (antidiabetic)	-↓ mitochondrial ATP production, ↓ oxygen consumption, ↑ lactate and glycolytic ATP production -↑AMPK, ↓ STAT3 & Akt/PKB -↓ SOX2 expression in TMZ-resistant glioma cells. (yang) -mTOR pathway inhibition -Inhibition of complex I of the ETC. -↓ superoxide dismutase (SOD) activity ↑ caspase 3 activity.	-Inhibition of STAT3 phosphorylation -↓ neurosphere formation. -↓ proliferation *via* chloride intracellular channel-1 (CLIC1) inhibition. G1 arrest. ↑AMPK, ↑FOXO3 – promotion of differentiation.	Leidgens et al. (196); Yang et al. (197); Gritti et al. (198); Owen et al. (199); Xiong et al. (200); Sato et al. (201)	NCT02780024	Metformin + radiation + TMZ.	Newly diagnosed GBM.	Estimated completion date December 2021.
				NCT03243851	Metformin + low dose TMZ.	Progressive or recurrent Glioblastoma.	No published results.
Chloroquine (antimalarial)	-Inhibition of autophagy. -Induction of p53-dependent apoptosis. Experimental indication of mitochondrial cristae damage and DNA break repair prevention (triple negative breast cancer stem cells).	-Inhibition of the PI3K/Akt pathway for sensitisation to radiation-induced apoptosis. -Inhibition of autophagy and promotion of apoptosis. -↑ radio sensitisation. Induction of p63-dependent G1 arrest.	Firat et al. (202); Kim et al. (203); Lee et al. (204); Ye et al. (205); Liang et al. (206)	NCT00224978	Chloroquine + conventional chemotherapy: caumustine and radiotherapy.	Tumour restricted to one brain hemisphere. First or second recurrence or relapse.	Median survival after surgery = 24 months for chloroquine-treated patients & 11 months for controls.
				NCT02432417	Chloroquine + chemoradiation	*de novo* GBM.	N/A. Estimated completion date January 2024.
				NCT02378532	Chloroquine + radiation +TMZ.	Newly diagnosed GBM, histopathological conformation of MGMT and EGFRvIII status.	44 adverse events recorded possibly/likely due to chloroquine including seizure and vomiting. Median overall survival = 8.1 months (EGFRvIII negative) & 13.4 months (EGFRvIII positive. 7 patients alive a median 9 month follow up. Maximum tolerated dose was established as 200mg daily chloroquine combined with RT and concurrent TMZ for newly diagnosed GBM.
Mefloquine (antimalarial)	Proposed cytotoxicity by inhibition of autophagy in glioma cells.		Golden et al. (207)	NCT01430351	Mefloquine + TMZ (arm 2 of study) + metformin (arm 6) + memantine (arm 4) + metformin & memantine (arm 7).	Histologically proven supratentorial glioblastoma.	Mefloquine induction of Abnormal ECG (2 patients) & QTc interval prolongation (1 patient). Possible induction of grade 1 sinus bradycardia (1 patient). Established final mefloquine dose - 250 mg 3 times weekly.
							Median OS = 21 months (95% CI, 16.2‐29.7 months), 2‐year survival rate = 43% (95% CI, 34%‐56%).
Atorvastatin (statin)	-Mevalonate pathway inhibition (leukaemia). -Inhibition of protein prenylation *via* upstream HMG-CoA reductase inhibition. -Suppression of Ras and downstream signalling pathways including Erk enhancement of TMZ efficacy.	-Apoptotic induction, ↓ migration and invasion of spheroids (U87).	Bayat et al. (208); Peng et al. (209)	NCT02029573	Atorvastatin + TMZ + radiation.	Histologically proven newly diagnosed GBM malignant GBM or variants. No prior chemotherapy or radiotherapy.	Interim analysis reported treatment safety, PFS-6 rate was 67% with median 9.1 months PFS. No published results from completed trial.
Celecoxib (NSAID)	-P53 dependent Induction of DNA damage and inhibition of DNA synthesis. Indications of G1 cell cycle arrest and autophagy.	-Regulation of chemokine axes (CCL2/CCR2 and CXCL10/CXCR3). Decrease of mRNA expression to viability of GSCs. -↓ of PD-L1 *via* FKBP5.	Kang et al. (210); Shono et al. (211); Yamaguchi et al. (212)	NCT00112502	Randomisation of 8 treatment arms. Combination of TMZ + thalidomide and/or celecoxib.	Histologically confirmed supratentorial GBM. Must have undergone biopsy, subtotal, or gross total tumour resection. Must have undergone radiotherapy within 5 weeks prior.	PFS for treatment arms combined was 11.6 months. Overall, 6-month PFS rate = 73%. Arms containing celecoxib showed a median PFS of 8.3 months compared with 7.4.
				NCT00068770	Two treatment arms: 1.p450 inhibitor + celecoxib + radiotherapy. 2.No p450 inhibitor + Celecoxib + radiotherapy.	Histologically confirmed GBM. No prior chemotherapy, radiotherapy, endocrine therapy, immunotherapy, or biological agents for malignancy. Recovered from surgery.	Study ended early, unethical to continue due to other trial data indicating the therapeutic benefit of including TMZ in treatment plan.
Disulfiram (alcohol addiction)	-Inhibition of ALDH. -Suppression of proteasomal activity. -↑ROS, activation of JNK & p38, inhibition of NF-κB in GBM cells. -Proposed modulation of apoptosis *via* Bcl2 family mediation. -Inhibition of PLK1 expression. -Inhibition of MGMT, ↑ alkylating DNA damage.	-Inhibition of chymotrypsin-like proteasomal activity, elevated effect with copper addition. -Inhibition of the ubiquitin-proteasome pathway. -Inhibition of self-renewal. -Enhancement of TMZ treatment activity in stem-cell like populations of GBM.	Hothi et al. (213); Triscott et al. (214); Paranjpe et al. (215)	NCT02678975	Disulfiram + copper + chemotherapy.	Previous diagnosis of glioblastoma (histologically verified) and presenting with a first progression/recurrence documented by MRI.	No published results.
				NCT01907165	Disulfiram + copper + TMZ.	Histologically confirmed GBM. Received or in the process of completing definitive radiotherapy with concurrent TMZ.	1-year PFS = 57%, & 1-year OS = 69%. No significant difference in PFS/OS according to Disulfiram dose, surgical extent, or MGMT methylation status. Better PFS & OS in GBMs with IDH1 (n = 6), BRAF (n = 2), or NF1 (n = 1) mutations than without: 1-year PFS: 100% *vs* 22%, respectively, p = 0.001; 1-year OS: 100% *vs* 42%, respectively, p = 0.006>
				NCT02715609	Preoperative treatment: Disulfiram + copper gluconate. Post-surgery: standard radiation + TMZ + concurrent Disulfiram/copper gluconate.	Dose escalation cohort: Diagnosis of GBM or its histological variants Dose expansion cohort: diagnosis of GBM (or its histological variants) with IDH, BRAF, or NF1 mutations. Confirmation of these mutations may be either by immunohistochemistry or next generation sequencing.	N/A. Estimated completion date December 15, 2023.
				NCT03034135	Disulfiram/copper gluconate + TMZ, 6-month course.	Histologically confirmed GBM, Radiotherapy completed with concurrent TMZ at least 12 weeks prior to start of study treatment. Or pathological verification of recurrent tumour at least 4 weeks after radiotherapy with concurrent TMZ. Exclusion of IDH mutants or secondary GBMs.	OOR = 0%, but 14% had clinical benefit. Median PFS = 1.7 months & median OS = 7.1 months. 1patient displayed dose limiting toxicity. Disulfiram concluded to have limited activity and was unable to recapitulate TMZ sensitivity in patients tested.

The potential mechanisms of these inhibitors against GSCs based on experimental studies are also included. The clinical trial identifiers are given as well as any published results from these studies.

## Conclusion

Mechanisms for conferring resistance to current GBM treatments have been described for GSCs including quiescence, slow cell cycling, upregulated expression of drug efflux proteins, enhanced DNA repair capacity, drug resistance and advanced plasticity. Attempting to overcome these molecular blockades has heightened the importance of delineating the metabolic phenotypes that are adopted by these cells; with shifts before, during and after standard treatment believed to be imperative for tumour reestablishment. metabolic inhibition strategies have recently gained traction, requiring a detailed knowledge of the metabolome and adaptability potential of cells following targeted pathway inhibition. However, it’s important to highlight that a metabolic treatment regimen designed to solely target GSCs may not improve GBM prognosis. For example, GSC differentiation induction strategies must ensure stable and terminal GSC differentiation as well as coordinated blocking of the reverse process. As highlighted by **Figure 1**, dedifferentiation of differentiated tumour bulk cells could result in the formation of new GSCs. Therefore, even if complete clearance of the GSC population could be achieved through metabolic targeting, this outcome may only be temporary, highlighting the limitations of this singular approach. In this way, metabolic regimens to target GSCs can be explored as an effective supplement for combinatorial use with current and emerging treatment strategies to ensure complete therapeutic coverage of heterogenic GBM tumours.

## Author Contributions

AH was responsible for article structure, figure design and building, drafting the introduction and main body of text. XL, MG, and MCG provided critical revision of the article and figures. CP provided critical revision of the article and figures, joint supervising author. KK was responsible for the article conception, drafting the article abstract and providing critical revision of the article and figures, joint supervising author. All authors contributed to the article and approved the submitted version.

## Funding

AH funded by Southmead Hospital Charity, North Bristol Trust Registered Charity No: 1055900. XL is funded by Cancer Research UK (grant number C30758/A2979).

## Conflict of Interest

The authors declare that the research was conducted in the absence of any commercial or financial relationships that could be construed as a potential conflict of interest.

## Publisher’s Note

All claims expressed in this article are solely those of the authors and do not necessarily represent those of their affiliated organizations, or those of the publisher, the editors and the reviewers. Any product that may be evaluated in this article, or claim that may be made by its manufacturer, is not guaranteed or endorsed by the publisher.
